# Dysfunctional Timing in Traumatic Brain Injury Patients: Co-occurrence of Cognitive, Motor, and Perceptual Deficits

**DOI:** 10.3389/fpsyg.2021.731898

**Published:** 2021-10-18

**Authors:** Laura Verga, Michael Schwartze, Sven Stapert, Ieke Winkens, Sonja A. Kotz

**Affiliations:** ^1^Research Group Comparative Bioacoustics, Max Planck Institute for Psycholinguistics, Nijmegen, Netherlands; ^2^Department of Neuropsychology and Psychopharmacology, Maastricht University, Maastricht, Netherlands; ^3^Zuyderland Medical Centre, Department of Medical Psychology, Sittard, Netherlands; ^4^Limburg Brain Injury Center, Maastricht University, Maastricht, Netherlands

**Keywords:** traumatic brain injury, timing, attention, sensorimotor synchronization, processing speed, SDMT = Symbol Digit Modalities Test, Digit Span

## Abstract

Timing is an essential part of human cognition and of everyday life activities, such as walking or holding a conversation. Previous studies showed that traumatic brain injury (TBI) often affects cognitive functions such as processing speed and time-sensitive abilities, causing long-term sequelae as well as daily impairments. However, the existing evidence on timing capacities in TBI is mostly limited to perception and the processing of isolated intervals. It is therefore open whether the observed deficits extend to motor timing and to continuous dynamic tasks that more closely match daily life activities. The current study set out to answer these questions by assessing audio motor timing abilities and their relationship with cognitive functioning in a group of TBI patients (*n* = 15) and healthy matched controls. We employed a comprehensive set of tasks aiming at testing timing abilities across perception and production and from single intervals to continuous auditory sequences. In line with previous research, we report functional impairments in TBI patients concerning cognitive processing speed and perceptual timing. Critically, these deficits extended to motor timing: The ability to adjust to tempo changes in an auditory pacing sequence was impaired in TBI patients, and this motor timing deficit covaried with measures of processing speed. These findings confirm previous evidence on perceptual and cognitive timing deficits resulting from TBI and provide first evidence for comparable deficits in motor behavior. This suggests basic co-occurring perceptual and motor timing impairments that may factor into a wide range of daily activities. Our results thus place TBI into the wider range of pathologies with well-documented timing deficits (such as Parkinson’s disease) and encourage the search for novel timing-based therapeutic interventions (e.g., employing dynamic and/or musical stimuli) with high transfer potential to everyday life activities.

## Introduction

Neurocognitive timing refers to the capacity to encode, decode, and process events in time and to temporally align with the environment (Grondin, 2010; Grondin et al., 2018). Although humans are sensitive to the timing of events across multiple timescales, the ability to process time in the milliseconds-to-seconds range is particularly relevant for perception, action, and cognition (Piras et al., 2014; Bader et al., 2019). Adequate timing abilities in this range constitute a precondition for everyday activities such as walking, holding a conversation, cooking, and for virtually any kind of goal-directed behavior. Conversely, even subtle impairments in timing abilities may have a profound impact on brain function, as evidenced by perceptual, cognitive, and motor symptoms arising from timing deficits in otherwise seemingly unrelated pathologies (e.g., Attention Deficit Hyperactivity Disorder, Toplak and Tannock, 2005; Parkinson’s Disease, PD, Benoit et al., 2014; Autism Spectrum Disorder, Falter and Noreika, 2011; Falter et al., 2011; Allman and Falter, 2015). This converging evidence sparked a growing interest in timing abilities as a possible cause for cognitive deficits in various neurological conditions and to develop novel therapeutic intervention strategies with a high transfer potential to numerous cognitive functions and, ultimately, daily life activities. For example, a music-based training program that employs rhythmic auditory cueing has proven effective in ameliorating both impaired motor and non-motor timing abilities in Parkinson’s Disease (Nombela et al., 2013; Benoit et al., 2014; Kotz and Gunter, 2015; Dalla Bella, 2018).

Traumatic Brain Injury (TBI) refers to an acute brain injury, resulting from a blow to the head caused by external physical forces, for instance traffic collisions, falls, or violence (Carroll et al., 2004; Andriessen et al., 2010), often leading to death or lifelong disability (Maas et al., 2008; Roozenbeek et al., 2013). TBI is categorized as mild, moderate, or severe based on acute injury characteristics (e.g., loss of consciousness, post-traumatic amnesia, brain damage). Next to neurodegenerative and developmental diseases, timing can also be compromised by TBI (Pouthas and Perbal, 2004; Mioni et al., 2013a; Piras et al., 2014; Bader et al., 2019); for example, some patients report difficulties in putting events in correct chronological order (e.g., while cooking). Across all levels of severity, timing impairments and cognitive symptoms–including extreme fatigue, impaired attention, working memory, and processing speed (Wallesch et al., 2001; Hoskison et al., 2009; Witt et al., 2010; Ghajar and Ivry, 2015)–are persistent and significantly hampered in daily life (Schretlen and Shapiro, 2003; Langlois et al., 2006; Barwood and Murdoch, 2013). As these symptoms tend to co-occur, it is challenging to disentangle their relative importance and causal relation (Piras et al., 2014; Bader et al., 2019). On the one hand, timing deficits may underlie and explain the seemingly varied cognitive symptoms observed in TBI (e.g., Ghajar and Ivry, 2008); on the other, several empirical studies suggest that cognitive deficits may be the cause of more elusive timing impairments (Perbal et al., 2003; Mioni et al., 2012, 2013b, 2014). The fact that cognitive impairments may be responsible for timing deficits is based on higher variability, but not necessarily lower performance accuracy, in TBI patients than healthy controls in perceptual tasks (e.g., Anderson and Schmitter-Edgecombe, 2011; Piras et al., 2014; for a review see Mioni et al., 2014); in addition, motor timing appears to be largely unaffected (Bader et al., 2019). Taken together, this evidence seems to downsize the relevance of timing in TBI. Importantly, however, most of these studies employed timing tasks targeting stimulus durations between 4 and 60 s, often limited to the presentation of isolated time intervals (for a review see Mioni et al., 2013a). This choice likely over-emphasizes cognitive deficits as timing in this range hinges on memory and executive functions, as opposed to sub-second time intervals that are assumed to be processed more automatically (Wing and Kristofferson, 1973a). In addition, the focus on isolated intervals stands in stark contrast to the dynamic nature of most daily activities. This distinction inspires the choice of timing tasks used in other pathologies, for example PD, that typically include continuous perception (e.g., beat processing; O’Boyle et al., 1996) and continuous production tasks (e.g., paced and unpaced finger-tapping) that often require participants to adapt to changing stimulus timing. The flexibility required for these tasks depends on both automatic as well as higher-level cognitive processes; for example, compensation for relatively subtle motor timing errors may require attention and awareness in response to unexpected tempo changes, but it can also be largely automatic for more predictable sequences (Mates, 1994; Repp and Keller, 2004). Thus, not only are dynamic tasks targeting sub-second intervals closer to everyday activities, but they may also differentiate the relationship between timing and cognitive impairments.

The empirical question whether to focus on single interval or continuous timing also impacts theoretical accounts of the neural mechanisms engaged in the processing of isolated intervals as opposed to the processing of sequential intervals. Mechanisms underlying isolated interval processing are mainly discussed in the context of “internal clock models” such as the seminal scalar expectancy theory (SET; Gibbon, 1977; Church, 1984, 2003; Gibbon and Allan, 1984; Gibbon et al., 1984). These models postulate the existence of an internal pacemaker and a switch-accumulator component (Wing and Kristofferson, 1973b; Vorberg and Wing, 1996; Mioni et al., 2013a; McAuley and Fromboluti, 2014) that may rely on the functioning of fronto-striatal and thalamic connections (Meck and Benson, 2002; Buhusi and Meck, 2005). Conversely, some models of continuous timing such as dynamic attending theory (DAT; Jones and Boltz, 1989) focus on the entrainment of endogenous oscillatory activity through external rhythms, potentially without the need for a localized central clock mechanism (McAuley et al., 2006; Grondin, 2010; Honing et al., 2018). Instead, this model relies on a distributed network responsible for large scale oscillatory activity that is tightly linked to attention (e.g., Large et al., 2015; Grondin et al., 2018). Interestingly, areas and connections belonging to such integrated timing network (including prefrontal, parietal areas, and cerebellum; Inglese et al., 2005; Kinnunen et al., 2011; Schwartze and Kotz, 2013; Eierud et al., 2014; Xiong et al., 2014) are known to be commonly affected by post-traumatic diffuse axonal injury (DAI; Messé et al., 2011; Shenton et al., 2012). DAI denotes the shearing and tearing of white matter tracts that is frequently caused by TBI and has emerged as an explanation for post-traumatic symptoms persisting in the absence of clear radiological evidence of brain lesions and independent of TBI severity (Shenton et al., 2012). Because of its diffuse influence on the timing network and its independence from TBI severity, DAI may explain the variability and variety of timing symptoms in this patient population.

The current study therefore set out to investigate timing perception and production, and their relationship with cognitive measures (e.g., processing speed, attention, working memory) in a heterogenous group of TBI patients. We expected to confirm perceptual timing impairments of previous studies and then to explore patients’ performance in more ecologically valid dynamic timing tasks (e.g., adaptive finger tapping). We hypothesized that these continuous tasks would lead to worse performance in TBI patients (e.g., higher tapping variability and/or lower adaptation indexes) as they rely on a large-scale timing network that is likely to be affected by DAI irrespective of TBI location and severity.

## Materials and Methods

### Participants

15 patients (6F, mean age 47.53 ± 14.05 years, range: 17–64 years, group: TBI) were recruited by a neuropsychologist (SZS) at Zuyderland Medical Centre (Sittard-Geleen, Netherlands). All patients suffered from a TBI of varied severity (from mild to severe) and were, at the time of testing, in a chronic phase post-injury (mean time since injury 4.98 ± 5.23 years, range: 5 months–19 years; Table 1). Exclusion criteria were: (i) history of multiple traumatic brain injuries, (ii) acute phase of the pathology, (iii) perinatal brain injury (i.e., the injury occurred before, during, or shortly after birth), (iv) presence of comorbid medical or psychiatric conditions, (v) hearing difficulties not corrected by hearing aids, (vi) motor impairments preventing the use of the index finger of the dominant hand. 15 healthy controls (5F, mean age 47.53 ± 12.53, range: 23–63 years, group: HC = Healthy Controls) were recruited from a participant database at Maastricht University (UM) to individually match the patient sample as closely as possible for age, gender, and education. All controls had normal hearing and no concurrent neurological or psychiatric conditions. All participants were right handed except for one left-handed patient and one control subject. Participants were compensated for travel costs to reach the testing facilities and gave their written informed consent before participating in the study. The study was approved by the local ethical committee of Maastricht University (Maastricht, Netherlands; agreement number: Master_184_07_10_2017/A1).

**TABLE 1:** Demographic characteristics of the patient sample and scores in the cognitive tests.

Patient	Age	Gender	Time since injury	Education	Severity	SDMT	DS-F	DS-B
0539	35	M	3 years	Middle	Severe	52	11	7
7182	61	M	6 years	High	Severe	45	16	9
0667	55	M	2 years	Low	Moderate	35	7	4
1149	47	F	6 years	Low	Severe	45	8	7
1670	17	F	4 years	Middle	Moderate	50	12	16
2515	46	F	1 years	Low	Mild	45	3	6
2848	64	M	6 months	Low	Severe	40	12	4
4383	39	F	7 years	High	Mild	45	6	5
5431	57	M	2 years	Middle	Severe	20	4	6
5719	55	M	19 years	Low	Severe	55	9	4
6303	46	F	8 years	Low	Moderate	35	7	9
7452	64	M	2 years	High	Severe	60	10	11
7860	58	F	13 years	High	Moderate to severe	50	13	12
8750	24	M	9 months	Middle	Moderate	40	8	7
9427	45	M	5 months	Middle	Mild	70	10	17

*M, Male; F, female; SDMT, Symbol Digit Modality Test; DS-F, Digit-Span, forward presentation; DS-B, Digit-Span, backward presentation.*

### Procedure

The study took place in a quiet testing room at Maastricht University to avoid distraction and contamination from external noise. The testing procedure involved: (i) an initial interview about each patient’s medical condition (i.e., diagnosis, time since injury, current medication, comorbidity with medical or psychiatric conditions; see Supplementary Material), handedness assessment (Edinburgh Handedness Inventory; Oldfield, 1971), and music expertise; (ii) the Symbol Digit Modalities Substitution Test (SDMT; Smith, 1982) to measure impairments of attention and processing speed following TBI (Bruijel et al., 2018); (iii) the Digit Span test (Wechsler, 2008) in both forward (DS-F) and backward (DS-B) forms to obtain a reliable indication of working memory and memory span; (iv) a series of tasks from the Battery for the Assessment of Auditory Sensorimotor and Timing Abilities (BAASTA; Dalla Bella et al., 2017; Bégel et al., 2018) to investigate participants’ perceptual and sensorimotor synchronization abilities. The selected tasks included:

• *Duration discrimination*: Participants listened to tone pairs (frequency = 1 kHz) to judge whether the second tone (comparison duration, range = 600–1,000 ms) lasted longer than the first (standard duration, 600 ms).
• *Anisochrony detection*: Participants judged whether sequences of 5 tones (1,047 Hz, tone duration = 150 ms, Inter-Onset Interval (IOI) = 600 ms) were isochronous (i.e., with a constant IOI) or not (i.e., the 4th tone was presented earlier than expected by up to 30% of the IOI).
• *Unpaced tapping*: To obtain a measure of preferred tapping rate and its variability, participants were asked to tap regularly at their most natural (self-chosen) rate for 60 s. This task was administered at the beginning and at the end of the BAASTA testing session to control for changes in spontaneous motor tempo due to the battery itself (e.g., induced tiredness), with the left and the right hand.
• *Paced Tapping*: Participants’ ability to synchronize with a metronome (i.e., an isochronous sequence of tones) was assessed by asking them to tap with their dominant index finger to a sequence of 60 piano tones (frequency = 1,319 Hz, IOI = 600 ms).
• *Synchronization continuation*: Participants were asked to tap with their dominant index finger in synchrony with an isochronous sequence of 10 tones (IOI = 600 ms) and to keep tapping for a duration corresponding to 30 IOIs of the pacing stimulus after the pacing ceased. Each trial at a given tempo was repeated twice.
• *Adaptive tapping*: To assess the ability to adapt to a tempo change in a synchronization-continuation task, participants tapped with their dominant index finger to an isochronous sequence of 10 tones. In 40% of the trials, the tempo of the last 4 tones could either increase, decrease, or remain constant (30 or 75 ms tempo change, i.e., the sequence IOI was adjusted by adding or subtracting 30 or 75 ms); in the remaining 60% of the trials the tempo remained constant (i.e., no IOI change). Participants were asked to adapt their tapping to the tempo change, and to keep tapping at the new tempo after the end of the sequence for a time corresponding to 10 IOIs. After each trial, participants judged whether they perceived a change in stimulus tempo (acceleration, deceleration, or no change). Trials were divided into 10 experimental blocks each including 6 trials (4 with tempo change, 2 without), presented in random order.

The first two tasks tested perceptual timing, while the remaining four targeted motor timing behavior. In each perceptual task, participants performed three blocks of 16 trials. Except for spontaneous tapping, all tasks were preceded by a practice trial. Please see Dalla Bella et al. (2017) for the tasks’ details. All BAASTA tasks were implemented as an app on one of two identical Samsung Galaxy TAB A6 tablet devices running Android 7.0 (see also e.g., Rathcke et al., 2021). Auditory stimuli were delivered via headphones (Sennheiser HD201) at a comfortable sound level. Participants tapped directly on the surface of the tablet. The testing procedure was identical for patients and healthy controls in the above order, except for the initial interview, which was not conducted with the control group.

### Data Analysis

#### BAASTA Perceptual Tasks

The thresholds for the duration discrimination and anisochrony detection tasks were obtained by averaging the values obtained in three blocks, expressed as percentages of the standard duration (Weber fraction). Blocks were rejected when they contained more than 30% of false alarms (i.e., participants incorrectly identified a difference in a no-difference trial).

#### BAASTA Production Tasks

Mean inter-tap intervals (ITIs) and their coefficient of variation (CV, obtained by dividing the ITIs SD by the mean) were calculated for each production task (see Dalla Bella et al., 2017; Bégel et al., 2018; for details on data pre-processing). For the paced tapping task, synchronization accuracy was calculated as the mean absolute asynchrony between taps and the respective pacing signal, while the corresponding standard error denoted synchronization variability. For the adaptive task, several further measures were calculated. First, the overall adaptation index (i.e., a measure of the quality of the tapping adaptation following the tempo change in the continuation phase) was calculated (Repp and Keller, 2004; Schwartze et al., 2011) for accelerations (i.e., final sequences with IOI < 600 ms—adaptation index acceleration) and decelerations (i.e., final sequences with IOI > 600 ms—adaptation index deceleration). To this end, regression lines were fitted to the slopes of the ITI functions of the final sequence tempo, and their slopes were used as adaptation indices with values of 1 indicating perfect adaptation, values lower than 1 indicating under correction, and values greater than 1 overcorrection. Second, phase correction and period correction were calculated based on Repp and Keller (2004; see also Mates, 1994); these indexes inform automatic (phase correction) vs. conscious and attentive (period correction) processes underlying adaptation to tempo changes. Third, the sensitivity index (d′) for detecting tempo changes was calculated based on the number of Hits (i.e., correct detection of a tempo change in either direction) and False alarms (i.e., incorrect detection of a tempo change). Extreme outliers in each task were defined as data points falling below the Q1 – 3^∗^Interquartile range (IQR) or above the Q3 + 3^∗^IQR, where Q1 is the first quartile and Q3 is the third quartile, and excluded.

### Statistical Analyses

Statistical analyses were conducted in Rstudio (RStudio Team, 2020, Version 1.3.959; R Studio Inc., Boston, United States) supporting R Version 4.0.2 (R Core Team, 2020).^1^ SDMT and DS scores were first corrected with the available norm scores based on education level (SDMT) and age (DS). Each dependent variable was inspected, and extreme within-group outliers (see BAASTA production task for details) were removed, while mild outlier values were retained (Bakker and Wicherts, 2014). Data were further checked for normality of the distribution by means of QQplots and Shapiro-Wilk tests (package *stats*), and for equality of variance between the groups (Levene’s test; package *car*) where appropriate. As most distributions significantly departed from normality, the Wilcoxon-Mann-Whitney test (package *stats*) was employed for group comparisons to reduce Type I errors and improve power even in the presence of mild outlier values (Bakker and Wicherts, 2014). Effect sizes are reported for significant tests as r with bootstrapped confidence intervals based on 1,000 replications (package *rcompanion;* Supplementary Table 3). To further corroborate the strength of significant results, we calculated and additionally reported *p*-values obtained from an n-1 jackknife resampling analysis (Tukey, 1958; package bootstrap). Spearman’s rank correlation coefficients (package *stats*) were calculated among variables showing significant group differences to evaluate the relation between cognitive scores and timing performance. Bonferroni correction was applied, when necessary, to account for multiple comparisons. All results were deemed significant at an alpha level of *p* < 0.05, two-sided.

## Results

### Cognitive Tasks

#### Symbol Digit Modalities Test

**FIGURE 1:**
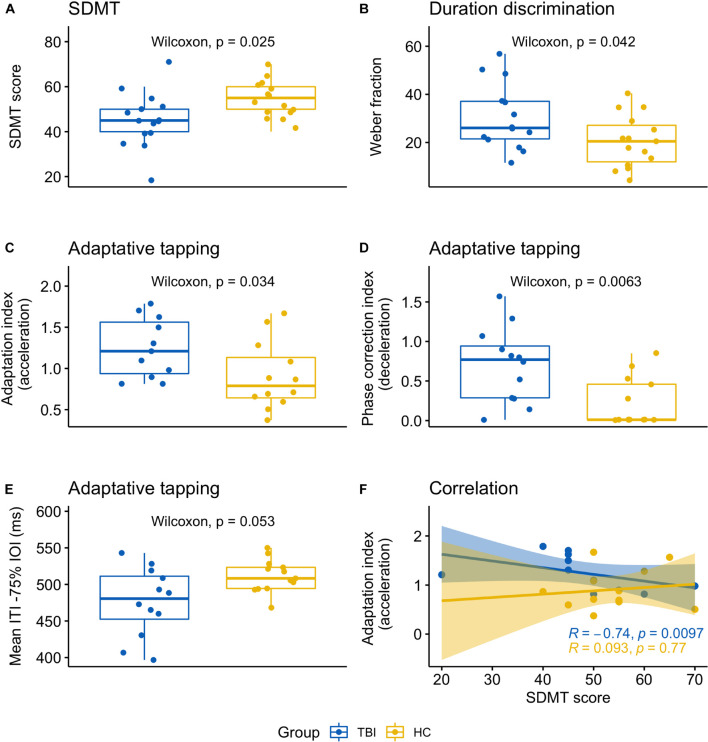
Graphical summary of significant results indicating the main differences between TBI patients and healthy controls. **(A)** SDMT scores; **(B)** Weber fraction corresponding to the duration discrimination threshold; **(C)** adaptive tapping task, adaptation index (acceleration); **(D)** Adaptive tapping task, phase correction index (deceleration); **(E)** adaptive tapping task, mean ITI (tapping rate) for accelerating sequences (i.e., reduction of 75 ms on the standard IOI); **(F)** correlation between the adaptation index (acceleration) and the SDMT scores.

TBI patients performed at an average score of 45.67 ± 11.63, which is at the lower normality boundary. This result was significantly lower than for healthy controls (mean_HC_ 54.00 ± 8.06; *W* = 58.50, *p* = 0.025, *r* = −0.41; Figure 1A) as confirmed by the jackknife estimate *p*-value (0.029 ± 0.01). Levene’s test was non-significant (*p* = 0.502) despite two patients who had slightly higher (patient 9,427 = 70) or lower (patient 5431 = 20) scores compared to the rest of their group.

#### Digit-Span, Forward Presentation

Patients’ scores were on average lower (mean_TBI_ 9.07 ± 3.47) than in healthy controls (mean_HC_ 10.53 ± 3.31), but the two groups had similar variance (Levene test *p* = 0.792), and did not differ statistically (*W* = 86.5, *p* = 0.287, *r* = −0.20).

#### Digit-Span, Backward Presentation

There was no significant difference between patients and controls (mean_TBI_ 8.27 ± 4.13; mean_HC_ 9.27 ± 2.49; *W* = 82, *p* = 0.209, *r* = 1, Levene test *p* = 0.171). One patient (patient 9427) obtained a moderately higher score (17); notably, patient 9427 had higher scores in the SDMT task as well.

### Perceptual Timing Tasks

#### Duration Discrimination

Data of one patient (patient 1149) were discarded as the threshold could not be reliably estimated (see Supplementary Table 2 for an overview of extreme or missing values in each task). The discrimination threshold was higher for TBI patients (mean_TBI_ 30.51 ± 13.72) than for healthy controls (mean_HC_ 20.51 ± 10.73). The difference between the two groups was statistically significant (*W* = 152, *p* = 0.042, *r* = 0.37; Levene’s test *p* = 0.485) as further confirmed by the jackknife analysis (mean *p*-value = 0.049 ± 0.02) (Figure 1B).

#### Anisochrony Detection

The discrimination threshold could not be determined for two patients and one control (patient 4,383, patient 6,303, control,8813; Supplementary Table 2). In addition, one patient (1,149) had a moderately higher threshold compared to the rest of the group (mean_#__1149_ 28.77 vs. mean_TBI_ 13.07 ± 7.63). Notably, for this patient there was also no valid estimation of the Duration Discrimination threshold. Yet, there was no significant difference between TBI and healthy controls (mean_HC_ 12.91 ± 7.33; *W* = 90.5, *p* = 1, *r* = 0.00; Levene’s test *p* = 0.953).

### Sensorimotor Timing Tasks

#### Unpaced Tapping

This task was performed at different testing times (beginning and end of the battery) and with different effectors (left and right hand). Thus, we performed an ANOVA with the between factor *group* and the within factors *effector* (left, right) and *time* (initial, final) for mean ITI and their CV. A summary of patients and controls who displayed extreme or missing values is reported in Supplementary Table 2. Mean ITIs were influenced by *effector* [*F*(1, 26) = 9.64, *p* = 0.005, η^2^ = 0.003]; *post hoc* tests revealed that the interval between taps was longer when the task was done with the right hand (mean_right_ 562.15, *SE* = 31.16; mean_left_ 542.97 ± 31.16; *t* = −3.10, *p* = 0.005). In addition, the interaction between time and effector reached significance [*F*(1, 26) = 4.75, *p* = 0.039, η^2^ = 0.001]: a simple effects analysis revealed no difference between left and right hand during the initial task (*p* = 0.84) but a significant difference in the final task (*t* = −3.76, *p* = 0.002, Tukey corrected; left hand t2 = 509.96, *SE* = 34.28; right hand t2 = 541.42, *SE* = 34.28). No other effects were significant (all ps > 0.07). Variability (CV_iti_) was not significantly influenced by any of the factors (all *p* > 0.07). However, variance between the TBI group and healthy controls was significant [Levene’s test: *F*(1, 27) = 5.21, *p* = 0.03], showing a generally higher variability in TBI patients.

#### Paced Tapping

Mean ITIs were very similar for TBI (mean_TBI_ 600.26 ± 1.55) and controls (mean_HC_ 599.80 ± 0.54; *W* = 85, *p* = 0.255, *r* = 0.21). Levene’s test was close to significance (*p* = 0.051), indicating slightly higher variance in the patients’ group. Motor variability was not significantly different between patients and controls (mean_TBI_.15 ± 0.17; mean_HC_.05 ± 0.01; *W* = 143, *p* = 0.102, *r* = 0.30), yet Levene’s test was (*p* = 0.035), indicating greater variance in patients. Synchronization accuracy (i.e., mean absolute asynchronies) was, on average, higher in the TBI group (mean_TBI_ 10.74 ± 7.18) than in the control group (mean_HC_ 8.67 ± 4.91), but this numerical difference was also not significant (*W* = 126, *p* = 0.590, *r* = 0.10). Lastly, synchronization variability (i.e., SE of asynchrony between tap and pacing signal), albeit higher in TBI (mean_TBI_ 1.14 ± 0.94) than in controls (mean_HC_ .77 ± 0.21), was not significantly different (*W* = 110, *p* = 0.369, *r* = 0.17).

#### Adaptive Tapping

The adaptation index (acceleration) was significantly different between patients and controls (mean_TBI_ 1.25 ± 0.36, mean_HC_ 0.91 ± 0.41; *W* = 101, *p* = 0.034, *r* = 0.39; average jackknife *p* = 0.04 ± 0.01; Figure 1C). In addition, the phase correction index for tempo decelerations was significantly lower for TBI compared to controls (mean_TBI_ .70 ± 0.47, mean_HC_ .22 ± 0.31; *W* = 127.50, *p* = 0.006, *r* = 0.50; Figure 1D). The jackknife estimate confirmed the robustness of this result (average jackknife *p* = 0.008 ± 0.001). The mean ITI in the slowest tempo condition (−75% IOI) was close to significance (*W* = 42, *p* = 0.053, *r* = −0.36; Figure 1E), with TBI patients tapping faster compared to controls (mean_TBI_ 476.03 ± 46.82; mean_HC_ 511.47 ± 22.24). Levene’s test was significant (*p* = 0.023), indicating different variance between the groups. Tapping variability (CV ITI) and judgment accuracy (d-prime) were not significant.

No other significant differences were observed in this task (all *p*s > 0.183).

#### Synchronization Continuation

There were no significant differences between patients and controls in any of the outcome variables for this task (all *p*s > 0.221).

### Correlations

We conducted a correlation analysis between SDMT and the timing tasks which significantly differed between groups. These included one perceptual task (duration discrimination) and two variables from the adaptive task (adaptation index acceleration, phase correction index deceleration) for a total of three repeated tests. When considering data from both groups, SDMT was not significantly correlated with any other measure; however, in the TBI group—but not in the healthy controls—a significant negative correlation emerged between SDMT and the adaptation index for accelerating sequences (*r*s = −0.74, *p* = 0.010; Bonferroni correction for multiple comparisons: 0.05/3 = 0.017; Figure 1F).

## Discussion

The current study set out to investigate timing abilities in TBI patients. We tested 15 patients in a series of timing perception and production tasks and compared their performance with that of age-, gender-, and education-matched healthy controls. Our results confirm functional impairments in the TBI group, affecting both cognitive as well as perceptual and motor timing abilities. Cognitively, patients showed deficits in processing speed, as indicated by lower scores in the SDMT compared to controls. In the timing domain, TBI patients displayed higher discrimination thresholds for sounds differing in duration. Patients also showed a reduced ability to adjust their finger tapping in response to tempo changes in auditory pacing sequences. This motor timing deficit further correlated with the SDMT score. Not only do our results confirm a clear impairment in processing speed and perceptual timing following TBI, but they also identify deficits in motor timing, which suggests a more generalized timing deficit than previously hypothesized in this pathology.

The results thus confirm previous evidence showing perceptual timing deficits in TBI (e.g., Mioni et al., 2013b) and higher performance variability in the patient group (Anderson and Schmitter-Edgecombe, 2011; Mioni et al., 2014; Piras et al., 2014). Overall increased variability in patients, in terms of a greater variance between groups, was evidenced by significant Levene’s tests in several tasks, namely unpaced, paced, and adaptive tapping. Notably, all these tasks tested motor timing, while group variance did not differ in either cognitive or perceptual timing tasks. These results therefore provide first important evidence of possible motor timing impairments. This is further supported by the finding that patients could not compensate for tempo changes in adaptive tapping. In this task, the adaptation index for accelerating sequences indicated a difficulty in “keeping up” with the pacing sequence. For decelerating sequences (i.e., sequences in which tempo was slowing down), patients had difficulties in applying phase correction to adapt to the changes. Yet, we must caution that the overall heterogeneity of the patient sample may be reflected in these results. Future studies need to further explore these aspects in larger and more homogeneous participant groups. Still, these results are particularly important for at least two reasons: first, motor timing has been typically considered as spared in TBI (Bader et al., 2019). For example, Perbal et al. (2003) found no motor deficit–but higher variability–in production or reproduction tasks in severe TBI (see also Pouthas and Perbal, 2004); similarly, Bader et al. (2019) found no evidence of motor timing deficits in mild TBI patients in a paced tapping task. Second, phase correction is considered a largely automatic process that is independent of cognitive functioning (Repp and Keller, 2004). By identifying this potentially “purer” timing deficit, the latter evidence speaks against the hypothesis that timing impairments may be the sole result of a failure in memory, attention, or other cognitive functions (Perbal et al., 2003; Mioni et al., 2012, 2013b, 2014).

It is possible that the adaptive task employed in the current experiment (Schwartze et al., 2011), may be more sensitive to subtle impairments as it targets the dynamic ability to flexibly recognize and adapt to tempo changes in continuous stimulation. As such, it may be better suited to more closely probe the timing requirements of daily life activities affected by TBI (e.g., wrapping a present; Schwartz et al., 1998). Alternatively, it is possible that previous studies failed to identify motor timing deficits as they exclusively targeted mild TBI (Bader et al., 2019; but see Perbal et al., 2003, for an example of severe patients). However, this explanation would stand in contrast with the hypothesis that an underlying DAI may affect the timing network, since DAI is assumed to be independent from TBI severity (Ghajar and Ivry, 2008; Shenton et al., 2012). Unfortunately, DAI can only be assessed by means of dedicated neuroimaging techniques such as Diffusion Tensor Imaging (DTI; Shenton et al., 2012). Future studies should therefore take advantage of DTI to evaluate the relationship between the extent of DAI and possible timing and cognitive deficits. The extreme variety observed in a typical TBI population, both in terms of symptoms and type of underlying lesion, calls for a highly individualized approach to identify possible patterns of co-occurring DAI and timing or cognitive deficits.

In the current study we observed deficits in processing speed, in line with previous accounts (e.g., Wallesch et al., 2001; Hoskison et al., 2009; Witt et al., 2010; Ghajar and Ivry, 2015). Most importantly, we report a correlation between SDMT scores and the adaptation index, reflecting lower adaptation abilities in patients with higher processing speed scores. This result stands in contrast with previous accounts showing that timing deficits go hand in hand with cognitive dysfunctions (e.g., Mioni et al., 2013b). While we clearly cannot use the current result to establish causality or independence between these measures, we suggest that timing and cognitive deficits in TBI may constitute a more complex relationship than previously hypothesized. Further, we suggest that dynamic timing tasks, closer matched to everyday life activities, may provide further insights into the complex symptomatology of this pathology.

Lastly, we put forward some limitations of the current study. First, our sample size was relatively small, due to the difficulty in patient recruitment. While our results are largely in line with the previous literature, a larger sample might help to strengthen some of our conclusions, particularly for those tasks (e.g., adaptive tapping) that are novel in the TBI literature. Hence, we highly encourage future studies to replicate our results within a larger group of participants. Second, we could not directly test specific hypotheses based on the neurological damage caused by TBI and/or DAI; as previously said, future studies should consider introducing neuroimaging (e.g., DTI) to further characterize the relationship between neurological (e.g., location and extent of the lesion, white matter integrity, etc.), cognitive, and timing deficits in TBI. A better characterization of individual deficits may allow informing individualized intervention strategies that consider each patient’s residual abilities as well as recovery potential. Third, our patient sample was quite heterogeneous. While we selected the patients in the current study to test for specific timing impairments irrespective of TBI severity levels, it is possible that heterogeneity contributed to the observed increased variability in most tasks in the patient group. Future studies should address this possibility by targeting several patient groups, a larger sample size, and more homogeneous level of TBI severity.

## Conclusion

In conclusion, the current study found evidence of cognitive, perceptual, and motor timing deficits in TBI patients. As a next step, we suggest that future studies should systematically explore the complex relationship between TBI symptoms and their underlying neurological causes. In doing so, it would be important to consider possible therapeutic approaches; in particular, we propose that timing, due to its fundamental role in everyday life, might represent an overarching principle with great potential for therapeutic impact, mirroring its application in other pathologies such as PD (Benoit et al., 2014; Dalla Bella, 2018).

## Data Availability Statement

The raw data supporting the conclusions of this article will be made available by the authors, without undue reservation.

## Ethics Statement

The studies involving human participants were reviewed and approved by the Ethical committee of Maastricht University, faculty of Psychology and Neuroscience (Maastricht, The Netherlands; agreement number: Master_184_07_10_2017/A1). The patients/participants provided their written informed consent to participate in this study.

## Author Contributions

MS and SK developed the theoretical framework. LV analyzed the data and wrote the manuscript. SS and IW recruited the participants and coordinated data collection. All authors conceived and developed the experiment and provided critical feedback on the research, analysis and manuscript.

## Conflict of Interest

The authors declare that the research was conducted in the absence of any commercial or financial relationships that could be construed as a potential conflict of interest.

## Publisher’s Note

All claims expressed in this article are solely those of the authors and do not necessarily represent those of their affiliated organizations, or those of the publisher, the editors and the reviewers. Any product that may be evaluated in this article, or claim that may be made by its manufacturer, is not guaranteed or endorsed by the publisher.

## Footnotes

1
http://www.R-project.org/

## References

[B1] Allman M. J. Falter C. M. (2015). “Abnormal timing and time perception in autism spectrum disorder?,” in *Time Distortions in Mind*, eds Vatakis A. Allman M. J. (Leiden: Brill), 37–56.

[B2] Anderson J. W. Schmitter-Edgecombe M. (2011). Recovery of time estimation following moderate to severe traumatic brain injury. *Neuropsychology* 25 36–44. 10.1037/a0020333 20919767 PMC3018715

[B3] Andriessen T. M. J. C. Jacobs B. Vos P. E. (2010). Clinical characteristics and pathophysiological mechanisms of focal and diffuse traumatic brain injury. *J. Cell Mol. Med.* 14 2381–2392. 10.1111/j.1582-4934.2010.01164.x 20738443 PMC3823156

[B4] Bader F. Kochen W. R. Kraus M. Wiener M. (2019). The dissociation of temporal processing behavior in concussion patients: stable motor and dynamic perceptual timing. *Cortex* 119 215–230. 10.1016/j.cortex.2019.04.019 31158558

[B5] Bakker M. Wicherts J. M. (2014). Outlier removal, sum scores, and the inflation of the type i error rate in independent samples t tests: the power of alternatives and recommendations. *Psychol. Methods* 19 409–427. 10.1037/met0000014 24773354

[B6] Barwood C. H. S. Murdoch B. E. (2013). Unravelling the influence of mild traumatic brain injury (MTBI) on cognitive-linguistic processing: a comparative group analysis. *Brain Injury* 27 671–676. 10.3109/02699052.2013.775500 23611468

[B7] Bégel V. Verga L. Benoit C.-E. E. Kotz S. A. Dalla Bella S. (2018). Test-retest reliability of the battery for the assessment of auditory sensorimotor and timing abilities (BAASTA). *Ann. Phys. Rehabil. Med.* 61 395–400. 10.1016/j.rehab.2018.04.001 29709607

[B8] Benoit C. E. Dalla Bella S. Farrugia N. Obrig H. Mainka S. Kotz S. A. (2014). Musically cued gait-training improves both perceptual and motor timing in Parkinson’s disease. *Front. Hum. Neurosci.* 8:494. 10.3389/fnhum.2014.00494 25071522 PMC4083221

[B9] Bruijel J. Stapert S. Z. Vermeeren A. Ponsford J. L. van Heugten C. M. (2018). Unraveling the biopsychosocial factors of fatigue and sleep problems after traumatic brain injury: protocol for a multicenter longitudinal cohort Study. *JMIR Res. Protoc.* 7:e11295. 10.2196/11295 30348629 PMC6231738

[B10] Buhusi C. V. Meck W. H. (2005). What makes us tick? Functional and neural mechanisms of interval timing. *Nat. Rev. Neurosci.* 6 755–765. 10.1038/nrn1764 16163383

[B11] Carroll L. Cassidy J. D. Peloso P. Borg J. von Holst H. Holm L. (2004). Prognosis for mild traumatic brain injury: results of the who collaborating centre task force on mild traumatic brain injury. *J. Rehabil. Med.* 36 84–105. 10.1080/16501960410023859 15083873

[B12] Church R. M. (1984). Properties of the internal clocka. *Ann. N. Y. Acad. Sci.* 423 566–582. 10.1111/j.1749-6632.1984.tb23459.x 6588815

[B13] Church R. M. (2003). “A concise introduction to scalar timing theory,” in *Functional and Neural Mechanisms of Interval Timing*, ed. Meck W. H. (Boca Raton, FL: CRC Press/Routledge/Taylor & Francis Group), 3–22.

[B14] Dalla Bella S. (2018). Music and movement: towards a translational approach. *Neurophysiol. Clin.* 48 377–386. 10.1016/j.neucli.2018.10.067 30396753

[B15] Dalla Bella S. Farrugia N. Benoit C. E. Begel V. Verga L. Harding E. (2017). BAASTA: battery for the assessment of auditory sensorimotor and timing abilities. *Behav. Res. Methods* 49 1128–1145. 10.3758/s13428-016-0773-6 27443353

[B16] Eierud C. Craddock R. C. Fletcher S. Aulakh M. King-Casas B. Kuehl D. (2014). Neuroimaging after mild traumatic brain injury: review and meta-analysis. *NeuroImage Clin.* 4 283–294. 10.1016/j.nicl.2013.12.009 25061565 PMC4107372

[B17] Falter C. M. Noreika V. (2011). Interval timing deficits and abnormal cognitive development. *Front. Integr. Neurosci.* 5:26. 10.3389/fnint.2011.00026 21716645 PMC3116141

[B18] Falter C. M. Noreika V. Wearden J. H. Bailey A. J. (2011). More consistent, yet less sensitive: interval timing in autism spectrum disorders. *Q. J. Exp. Psychol.* 65 2093–2107. 10.1080/17470218.2012.690770 22800511

[B19] Ghajar J. Ivry R. B. (2008). The predictive brain state: timing deficiency in traumatic brain injury? *Neurorehabil. Neural Repair* 22 217–227. 10.1177/1545968308315600 18460693 PMC4338277

[B20] Ghajar J. Ivry R. B. (2015). The predictive brain state?: timing deficiency in traumatic brain. *Neurorehabil. Neural Repair* 22 217–227. 10.1177/1545968308315600 PMC4338277 18460693

[B21] Gibbon J. (1977). Scalar expectancy theory and Weber’s law in animal timing. *Psychol. Rev.* 84 279–325. 10.1037/0033-295X.84.3.279

[B22] Gibbon J. Allan L. (1984). *Timing and time perception. Annals of the New York Academy of Sciences.* New York, NY: New York Academy of Sciences.

[B23] Gibbon J. Church R. M. Meck W. H. (1984). Scalar timing in memory. *Ann. N.Y. Acad. Sci.* 423 52–77. 10.1111/j.1749-6632.1984.tb23417.x 6588812

[B24] Grondin S. (2010). Timing and time perception: a review of recent behavioral and neuroscience findings and theoretical directions. *Attent. Percept. Psychophys.* 72 561–582. 10.3758/APP.72.3.561 20348562

[B25] Grondin S. Hasuo E. Kuroda T. Nakajima Y. (2018). “Auditory time perception,” in *Springer Handbooks*, eds Siciliano B. Khatib O. (Berlin: Springer), 423–440.

[B26] Honing H. Bouwer F. L. Prado L. Merchant H. (2018). Rhesus Monkeys (*Macaca mulatta*) sense isochrony in rhythm, but not the beat: additional support for the gradual audio motor evolution hypothesis. *Front. Neurosci.* 12:475. 10.3389/fnins.2018.00475 30061809 PMC6054994

[B27] Hoskison M. M. Moore A. N. A. Hu B. Orsi S. Kobori N. Dash P. K. K. (2009). Persistent working memory dysfunction following traumatic brain injury: evidence for a time-dependent mechanism. *Neuroscience* 159 483–491. 10.1016/j.neuroscience.2008.12.050 19167462 PMC4264540

[B28] Inglese M. Makani S. Johnson G. Cohen B. A. Silver J. A. Gonen O. (2005). Diffuse axonal injury in mild traumatic brain injury: a diffusion tensor imaging study. *J. Neurosurg.* 103 298–303. 10.3171/jns.2005.103.2.0298 16175860

[B29] Jones M. R. Boltz M. (1989). Dynamic attending and responses to time. *Psychol. Rev.* 96:459. 10.1037/0033-295x.96.3.459 2756068

[B30] Kinnunen K. M. Greenwood R. Powell J. H. Leech R. Hawkins P. C. Bonnelle V. (2011). White matter damage and cognitive impairment after traumatic brain injury. *Brain* 134 449–463. 10.1093/brain/awq347 21193486 PMC3030764

[B31] Kotz S. A. Gunter T. C. (2015). Rhythmic cueing remediates language-related disorders in Parkinson’s disease. *Ann. N.Y. Acad. Sci.* 1337 62–68. 25773618 10.1111/nyas.12657

[B32] Langlois J. A. Rutland-Brown W. Wald M. M. (2006). The epidemiology and impact of traumatic brain injury. *J. Head Trauma Rehabil.* 21 375–378. 10.1097/00001199-200609000-00001 16983222

[B33] Large E. W. Herrera J. A. Velasco M. J. (2015). Neural networks for beat perception in musical rhythm. *Front. Syst. Neurosci.* 9:159. 10.3389/fnsys.2015.00159 26635549 PMC4658578

[B34] Maas A. I. Stocchetti N. Bullock R. (2008). Moderate and severe traumatic brain injury in adults. *Lancet Neurol.* 7 728–741. 10.1016/S1474-4422(08)70164-9 18635021

[B35] Mates J. (1994). A model of synchronization of motor acts to a stimulus sequence - II. Stability analysis, error estimation and simulations. *Biol. Cybernet.* 70 475–484. 10.1007/BF00203240 8186307

[B36] McAuley J. D. Fromboluti E. K. (2014). Attentional entrainment and perceived event duration. *Philos. Trans. R. Soc. B Biol. Sci.* 369:20130401. 10.1098/rstb.2013.0401 25385779 PMC4240968

[B37] McAuley J. D. Jones M. R. Holub S. Johnston H. M. Miller N. S. (2006). The time of our lives: life span development of timing and event tracking. *J. Exp. Psychol. Gen.* 135 348–367. 10.1037/0096-3445.135.3.348 16846269

[B38] Meck W. H. Benson A. M. (2002). Dissecting the brain’s internal clock: how frontal-striatal circuitry keeps time and shifts attention. *Brain Cogn.* 48 195–211. 10.1006/brcg.2001.1313 11812042

[B39] Messé A. Caplain S. Paradot G. Garrigue D. Mineo J. F. Soto Ares G. (2011). Diffusion tensor imaging and white matter lesions at the subacute stage in mild traumatic brain injury with persistent neurobehavioral impairment. *Hum. Brain Mapp.* 32 999–1011. 10.1002/hbm.21092 20669166 PMC6870077

[B40] Mioni G. Grondin S. Stablum F. (2014). Temporal dysfunction in traumatic brain injury patients: primary or secondary impairment? *Front. Hum. Neurosci.* 8:269. 10.3389/fnhum.2014.00269 24817847 PMC4012215

[B41] Mioni G. Mattalia G. Stablum F. (2013a). Time perception in severe traumatic brain injury patients: a study comparing different methodologies. *Brain Cogn.* 81 305–312. 10.1016/j.bandc.2012.12.005 23395855

[B42] Mioni G. Stablum F. Cantagallo A. (2013b). Time discrimination in traumatic brain injury patients. *J. Clin. Exp. Neuropsychol.* 35 90–102. 10.1080/13803395.2012.755151 23259647

[B43] Mioni G. Stablum F. McClintock S. M. Cantagallo A. (2012). Time-based prospective memory in severe traumatic brain injury patients: the involvement of executive functions and time perception. *J. Int. Neuropsychol. Soc.* 18 697–705. 10.1017/S1355617712000306 22433779

[B44] Nombela C. Hughes L. E. Owen A. M. Grahn J. A. (2013). Into the groove: can rhythm influence Parkinson’s disease? *Neurosci. Biobehav. Rev.* 37 2564–2570. 10.1016/j.neubiorev.2013.08.003 24012774

[B45] O’Boyle D. J. Freeman J. S. Cody F. W. J. (1996). The accuracy and precision of timing of self-paced, repetitive movements in subjects with Parkinson’s disease. *Brain* 119 51–70. 10.1093/brain/119.1.51 8624694

[B46] Oldfield R. C. (1971). The assessment and analysis of handedness: the Edinburgh inventory. *Neuropsychologia* 9 97–113. 5146491 10.1016/0028-3932(71)90067-4

[B47] Perbal S. Couillet J. Azouvi P. Pouthas V. (2003). Relationships between time estimation, memory, attention, and processing speed in patients with severe traumatic brain injury. *Neuropsychologia* 41 1599–1610. 10.1016/S0028-3932(03)00110-6 12887985

[B48] Piras F. Piras F. Ciullo V. Danese E. Caltagirone C. Spalletta G. (2014). Time dysperception perspective for acquired brain injury. *Front. Neurol.* 4:217. 10.3389/fneur.2013.00217 24454304 PMC3888944

[B49] Pouthas V. Perbal S. (2004). Time perception depends on accurate clock mechanisms as well as unimpaired attention and memory processes. *Acta Neurobiol. Exp.* 64 367–385. 10.55782/ane-2004-1520 15283479

[B50] R Core Team (2020). *R: A Language and Environment for Statistical Computing.* Vienna: R Foundation for Statistical Computing.

[B51] Rathcke T. Lin C.-Y. Falk S. Bella S. D. (2021). Tapping into linguistic rhythm. *Lab. Phonol. J. Assoc. Lab. Phonol.* 12:11.

[B52] Repp B. H. Keller P. E. (2004). Adaptation to tempo changes in sensorimotor synchronization: effects of intention, attention, and awareness. *Q. J. Exp. Psychol. Sec. A* 57 499–521. 10.1080/02724980343000369 15204138

[B53] Roozenbeek B. Maas A. I. R. Menon D. K. (2013). Changing patterns in the epidemiology of traumatic brain injury. *Nat. Rev. Neurol.* 9 231–236. 10.1038/nrneurol.2013.22 23443846

[B54] RStudio Team (2020). *RStudio: Integrated Development Environment for R.* Vienna: R Foundation for Statistical Computing.

[B55] Schretlen D. J. Shapiro A. M. (2003). A quantitative review of the effects of traumatic brain injury on cognitive functioning. *Int. Rev. Psychiatry* 15 341–349. 10.1080/09540260310001606728 15276955

[B56] Schwartz M. F. Montgomery M. W. Buxbaum L. J. Lee S. S. Carew T. G. Coslett H. B. (1998). Naturalistic action impairment in closed head injury. *Neuropsychology* 12 13–28. 10.1037//0894-4105.12.1.13 9460731

[B57] Schwartze M. Keller P. E. Patel A. D. Kotz S. A. (2011). The impact of basal ganglia lesions on sensorimotor synchronization, spontaneous motor tempo, and the detection of tempo changes. *Behav. Brain Res.* 216 685–691. 10.1016/j.bbr.2010.09.015 20883725

[B58] Schwartze M. Kotz S. A. (2013). A dual-pathway neural architecture for specific temporal prediction. *Neurosci. Biobehav. Rev.* 37 2587–2596. 10.1016/j.neubiorev.2013.08.005 23994272

[B59] Shenton M. E. Hamoda H. M. Schneiderman J. S. Bouix S. Pasternak O. Rathi Y. (2012). A review of magnetic resonance imaging and diffusion tensor imaging findings in mild traumatic brain injury. *Brain Imaging Behav.* 6 137–192. 10.1007/s11682-012-9156-5 22438191 PMC3803157

[B60] Smith A. (1982). *Symbol Digit Modalities Test (SDMT).* Los Angeles, CA: Western Psychological Services.

[B61] Toplak M. E. Tannock R. (2005). Time perception: modality and duration effects in attention-deficit/hyperactivity disorder (ADHD). *J. Abnorm. Child Psychol.* 33 639–654. 10.1007/s10802-005-6743-6 16195956

[B62] Tukey J. W. (1958). Bias and confidence in not-quite large samples. *Ann. Math. Stat.* 29 614–623. 10.1214/aoms/1177706647

[B63] Vorberg D. Wing A. (1996). “Chapter 4 Modeling variability and dependence in timing,” in *Handbook of Perception and Action*, eds Neumann O. Sanders A. F. (Cambridge, MA: Academic Press), 181–262.

[B64] Wallesch C. W. Curio N. Galazky I. Jost S. Synowitz H. (2001). The neuropsychology of blunt head injury in the early postacute stage: effects of focal lesions and diffuse axonal injury. *J. Neurotrauma* 18 11–20. 10.1089/089771501750055730 11200246

[B65] Wechsler D. (2008). *WAIS-IV?: Wechsler Adult Intelligence Scale.* London: Pearson.

[B66] Wing A. M. Kristofferson A. B. (1973a). Response delays and the timing of discrete motor responses. *Percept. Psychophys.* 14 5–12. 10.3758/BF03198607

[B67] Wing A. M. Kristofferson A. B. (1973b). The timing of interresponse intervals. *Percept. Psychophys.* 13 455–460. 10.3758/BF03205802

[B68] Witt S. T. Lovejoy D. W. Pearlson G. D. Stevens M. C. (2010). Decreased prefrontal cortex activity in mild traumatic brain injury during performance of an auditory oddball task. *Brain Imaging Behav.* 4 232–247. 10.1007/s11682-010-9102-3 20703959

[B69] Xiong K. L. Zhu Y. S. Zhang W. G. (2014). Diffusion tensor imaging and magnetic resonance spectroscopy in traumatic brain injury: a review of recent literature. *Brain Imaging Behav.* 8 487–496. 10.1007/s11682-013-9288-2 24449140

