# Supplementary material for: Dysfunctional Timing in Traumatic Brain Injury Patients: Co-occurrence of Cognitive, Motor, and Perceptual Deficits
Source: Front Psychol. 2021 Oct 18;12:731898. doi: 10.3389/fpsyg.2021.731898 (PMC8558219; doi:10.3389/fpsyg.2021.731898)
Supplement: Supplementary file 1 [file Data_Sheet_1.docx]

***Supplementary Material***

**1. Interview for TBI patients**

1. Heeft u ooit eerder een hersenletsel of beroerte opgelopen dan wel doorgemaakt?

*Have you ever had a brain injury or stroke?*

1. Wanneer heeft u het hersenletsel gekregen? Welke leeftijd had u? Hoe lang geleden is dat?

*When did you get the brain injury? What age were you? How long ago was it?*

1. Hoe ernstig was het letsel?

*How serious was the injury?*

1. Had u uw bewustzijn verloren? Zo ja, hoe lang?

*Had you lost consciousness? If so, for how long?*

1. Heeft u last gehad van cognitieve (geheugenverlies) of emotionele veranderingen? Zo ja, hoe lang?

*Have you suffered from cognitive (memory loss) or emotional changes? If so, for how long?*

1. Heeft u ooit een diagnose gekregen m.b.t. uw letsel?

*Have you ever been diagnosed with an injury?*

1. Neemt u momenteel nog medicatie voor uw hersenletsel? Zo ja, welke?

*Are you currently taking medication for your brain injury? If so, which ones?*

1. Heeft u therapie gekregen in het verleden? Zo ja, welke? En hoe lang?

*Have you received therapy in the past? If so, which ones? And for how long?*

1. Wat was het doel van de therapie? Welke disciplines?

*What was the purpose of the therapy? What kinds?*

1. Krijgt u nu nog steeds therapie? Zijn er nog hulpverleners betrokken?

*Are you still getting therapy? Are counsellors still involved?*

1. Heeft u andere klachten? Bv depressie?

*Do you have any other complaints? For example depression?*

1. Neemt u daar medicatie voor? Welke? Dosis?

*Do you take medication for that? What medication? Dose?*

1. Wat is uw hoogst behaalde diploma? In hoeveel jaar heeft u dit behaald?

*What is your highest degree? In how many years have you obtained it?*

1. Hoe is uw gezinssituatie? Bent u getrouwd? Heeft u kinderen?

*How's your family situation? Are you married? Do you have children?*

1. Heeft u muzikale expertise? Kan u een instrument spelen of heeft u ooit muziekles gevolgd? Vragenlijst over muzikale expertise

*Do you have musical expertise? Can you play an instrument or have you ever taken music lessons? (Musical Aptitude Questionnaire)*

1. Bent u links- of rechtshandig? vragenlijst

*Are you left-handed or right-handed? Oldfield Questionnaire*

**2. Additional tables**

**Table 2.** Summary of tasks in which the sample was reduced due to missing or extreme data values. Abbreviations: t1 = task performed at the beginning of the BAASTA battery; t2 = task performed at the end of the BAASTA battery.

|  | | | | | |
| --- | --- | --- | --- | --- | --- |
| **Task** | **Variable** | **TBI** | | **HC** | |
|  |  | **Subject** | **Value** | **Subject** | **Value** |
| Duration discrimination | Threshold | 1149 | NA |  |  |
| Anisochrony detection | Threshold | 4383  6303 | NA | 8813 | NA |
| Unpaced tapping | Mean ITI t1, left | 2848 | 2205.97 |  |  |
|  | Mean ITI t2, left | 667 | NA |  |  |
|  | CV ITI t1, right |  |  | 9226 | .61 |
|  | CV ITI t1, left | 667  2515  2848 | .46  .45  .79 | 2681  9226 | .55  .62 |
|  | CV ITI t2, right | 667  2848 | .38  .53 | 9123  9226 | .44  .59 |
|  | CV ITI t2, left | 667  2848 | NA  .43 |  |  |
| Paced tapping | Mean ITI | 539  9427  6352  2848 | 708.52  556.14  565.47  515.02 | 6352  8682  9226 | 603.02  598.39  773.50 |
|  | CV ITI |  |  | 9226 | .46 |
|  | SEM absolute asynchrony | 667  2848 | 8.41  7.48 | 9226 | 2.30 |
| Adaptive tapping | All sub-tasks | 667  1670  2848 | NA  NA  NA | 7424  9123 | Na  Na |
|  | Acceleration index | 6303 | 4.62 | 2681 | 4.42 |
|  | CV ITI |  |  | 2681  9226 | .44  .45 |
|  | +75 CV ITI |  |  | 9226 | .63 |
|  | - 30 mean ITI |  |  | 2681 | 667.05 |
|  | + 75 mean ITI | 6303 | 496.86 |  |  |
| Synchronization continuation | Mean ITI | 667  1670 | 692.97  719.11 | 9123 | Na |
|  | CV ITI | 667  1670  2848 | .59  .26  .40 | 9123  9226 | Na  .54 |

| **Table 3.** Summary of results (means and standard deviations) for each dependent variable and each task. P-values are reported for between-group results (Wilcoxon-Mann-Whitney test) with an estimate of the effect size (r with bootstrapped 95% confidence intervals based on 1000 replications). Significant results are indicated by a light grey background. | | | | | |
| --- | --- | --- | --- | --- | --- |
| **Task** | **Variable** | **TBI mean (SD)** | **HC mean (SD)** | **p** | **r**  **[ 95% CI ]** |
|  |  |  |  |  |  |
| SDMT | Corrected score | 45.67 (11.63) | 54.00 (8.06) | .025* | -.41  [-.70; -.10] |
| DS-F | Corrected score | 9.07 (3.47) | 10.53 (3.31) | .287 | -.20  [-.51; .15] |
| DS-B | Corrected score | 8.27 (4.13) | 9.27 (2.49) | .209 | 1  [-.23; -.60] |
| Duration Discrimination | ﻿Threshold (Weber fraction) | 30.51 (13.72) | 20.51 (10.73) | .042* | .37  [.02; .65] |
| Anisochrony Detection | Threshold (Weber fraction) | 13.07 (7.63) | 12.91 (7.33) | 1 | .00  [-.38; .35] |
| Unpaced Tapping | Inter-Tap Interval | t1, r: 529.93 (248.71) | t1, r: 608.24 (190.32) | .263 | -.21  [-.52; .18] |
|  |  | t1, l: 537.29 (252.68) | t1, l: 603.02 (199.19) | .348 | -.18  [-.52; .22] |
|  |  | t2, r: 521.03 (106.65) | t2, r: 549.89 (122.22) | .619 | -.09  [-.43; .28] |
|  |  | t2, l: 506.37  (157.30) | t2, l: 530.92  (106.31) | .616 | -.10  [-.45;.28] |
|  | Motor Variability (CV) | t1, r: .15 (.15) | t1, r: .06 (.01) | .111 | .29  [-.08; .59] |
|  |  | t1, l: .05 (.01) | t1, l: .05 (.01) | .978 | .01  [-.34; .39] |
|  |  | t2, r: .07 (.03) | t2, r: .06 (.02) | .356 | .17  [-.19; .53] |
|  |  | t2, l: .06 (.02) | t2, l: .06 (.02) | .790 | -.05  [-.45; .33] |
| Paced Tapping Metronome | Inter-Tap Interval | 600.26 (1.55) | 599.80 (.54) | .255 | .21  [-.22; .57] |
|  | Motor Variability (CV) | .15 (.17) | 0.05 (.01) | .102 | .30  [-.05; .62] |
|  | Mean absolute asynchrony | 10.74 (7.18) | 8.67 (4.91) | .590 | .10  [-.29; .48] |
|  | SE absolute asynchrony | 1.14 (.94) | 0.77 (.21) | .369 | .17  [-.23; .51] |
| Adaptive Tapping | *d’* (acceleration, -75% IOI) | 3.60 (.46) | 3.58 (.42) | .977 | .01  [-.34; .40] |
|  | *d’* (acceleration, -30% IOI) | 2.09 (.95) | 2.44 (.70) | .241 | -.22  [-.53; .17] |
|  | *d’* (deceleration, +30% IOI) | 2.51 (.92) | 2.45 (.74) | .643 | .09  [-.28; .45] |
|  | *d’* (deceleration, +75% IOI) | 3.65(.46) | 3.44 (.44) | .248 | .22  [-.16; .58] |
|  | Adaptation index (acceleration) | 1.25 (.36) | .91 (.41) | .034* | .39  [.08; .65] |
|  | Adaptation index (deceleration) | .95 (.58) | 1.22 (1.07) | .463 | -.14  [-.48; .25] |
|  | Phase correction index (acceleration) | .72 (.33) | .86 (.54) | .430 | -.15  [-.50; .24] |
|  | Phase correction index(deceleration) | .70 (.47) | .22 (.31) | .006** | .50  [.18; .73] |
|  | Period correction index (acceleration) | 1.02 (.45) | .97 (.67) | .624 | **.09**  **[-.32; .46]** |
|  | Period correction index (deceleration) | .81 (.59) | .82 (.85) | .913 | .02  [-.35; .40] |
|  | Inter-Tap Interval (no change) | 556.5 (39.96) | 582.37 (29.11) | .183 | -.25  [-.56; .11] |
|  | Inter-Tap Interval (-75% IOI) | 476.03 (46.82) | 511.47 (22.24) | .053° | -.36  [-.65, 0] |
|  | Inter-Tap Interval (-30% IOI) | 544.85 (43.31) | 555.88 (18.69) | .624 | -.09  [-.44;.29] |
|  | Inter-Tap Interval (+30% IOI) | 589.68 (44.53) | 594.12 (40.14) | .683 | .08  [-.29; .49] |
|  | Inter-Tap Interval (+75% IOI) | 644.92 (28.38) | 649.01 (40.37) | .954 | .02  [-.34; .41] |
|  | CV (no change) | .16 (.10) | .17 (.06) | .518 | -.12  [-.51; .28] |
|  | CV (-75% IOI) | .14 (.08) | .13 (.10) | .470 | .14  [-.20; .48] |
|  | CV (-30% IOI) | .15 (.11) | .17 (.11) | .605 | -.10  [-.45; .27] |
|  | CV (+30% IOI) | .19 (.13) | .19 (.12) | .765 | -.06  [-.43; .30] |
|  | CV (+75% IOI) | .18 (.08) | .19 (.13) | .624 | .09  [-.27; .45] |
| Synchronization Continuation | Inter-Tap Interval | 589.65 (20.88) | 605.52 (39.75) | .423 | -.15  [-.47; .20] |
|  | Motor Variability (CV) | .05 (.01) | .04 (.01) | .221 | .23  [-.10; .54] |
